# GPR55 deficiency is associated with increased adiposity and impaired insulin signaling in peripheral metabolic tissues

**DOI:** 10.1096/fj.201800171R

**Published:** 2018-08-27

**Authors:** Christopher Lipina, Sarah K. Walsh, Sharon E. Mitchell, John R. Speakman, Cherry L. Wainwright, Harinder S. Hundal

**Affiliations:** * Division of Cell Signalling and Immunology, Sir James Black Centre, School of Life Sciences, University of Dundee, Dundee, United Kingdom;; † Centre for Cardiometabolic Health Research, Robert Gordon University, Aberdeen, United Kingdom;; ‡ Institute of Biological and Environmental Sciences, University of Aberdeen, Aberdeen, United Kingdom;; § Institute of Genetics and Developmental Biology, Chinese Academy of Sciences, Beijing, China

**Keywords:** endocannabinoid, cannabinoid receptor, skeletal muscle, liver, adipogenesis

## Abstract

Emerging evidence indicates that G-protein coupled receptor 55 (GPR55), a nonclassic receptor of the endocannabinoid system that is activated by L-α-lysophosphatidylinositol and various cannabinoid ligands, may regulate endocrine function and energy metabolism. We examined how GPR55 deficiency and modulation affects insulin signaling in skeletal muscle, adipose tissue, and liver alongside expression analysis of proteins implicated in insulin action and energy metabolism. We show that GPR55-null mice display decreased insulin sensitivity in these tissues, as evidenced by reduced phosphorylation of PKB/Akt and its downstream targets, concomitant with increased adiposity and reduced physical activity relative to wild-type counterparts. Impaired tissue insulin sensitivity coincided with reduced insulin receptor substrate-1 abundance in skeletal muscle, whereas in liver and epididymal fat it was associated with increased expression of the 3-phosphoinoistide lipid phosphatase, phosphatase and tensin homolog. In contrast, GPR55 activation enhanced insulin signaling in cultured skeletal muscle cells, adipocytes, and hepatocytes; this response was negated by receptor antagonists and GPR55 gene silencing in L6 myotubes. Sustained GPR55 antagonism in 3T3-L1 adipocytes enhanced expression of proteins implicated in lipogenesis and promoted triglyceride accumulation. Our findings identify GPR55 as a positive regulator of insulin action and adipogenesis and as a potential therapeutic target for countering obesity-induced metabolic dysfunction and insulin resistance.—Lipina, C., Walsh, S. K., Mitchell, S. E., Speakman, J. R., Wainwright, C. L., Hundal, H. S. GPR55 deficiency is associated with increased adiposity and impaired insulin signaling in peripheral metabolic tissues.

Pathologic over activation of the endocannabinoid system (ECS) is associated with the development of dyslipidemia, obesity, and type II diabetes mellitus (1–4). Indeed, the ECS can act centrally and peripherally to regulate a number of metabolic processes, including feeding behavior (5), energy expenditure (6, 7), and nutrient uptake and metabolism (8–10). Key ECS components include the G-protein coupled receptors cannabinoid receptor (CB)1 and CB2 and their endogenous lipid ligands anandamide and 2-arachidonylglycerol, collectively known as endocannabinoids (11–14). Modulation of these receptors has been shown to promote significant alterations in body weight and various metabolic parameters in rodents and humans (15, 16). More specifically, CB1 receptor activation can result in insulin desensitization and suppressed metabolic activity, whereas inhibition of this same receptor has been shown to attenuate such manifestations of obesity-induced metabolic syndrome (6, 10, 17, 18).

In addition to activating CB1 and CB2, some cannabinoids (termed atypical cannabinoids) can promote cellular responses by acting through non-CB1/CB2 receptors such as the G-protein coupled receptor 55 (GPR55) (19). GPR55 is considered by some as a third member of the cannabinoid receptor family. However, this issue remains contentious because GPR55 shares little amino acid identity (∼14%) and lacks the classic “cannabinoid binding pocket” present in CB1 and CB2 (20). Nonetheless, the action of atypical cannabinoids at GPR55 has attracted significant attention because the receptor is expressed widely within the body but also because of evidence linking GPR55 modulation to regulation of diverse physiologic and pathologic processes, including endocrine function, tissue inflammation, and energy metabolism (21–23). For example, 5-methyl-4-[(1R,6R)-3-methyl-6-(1-cyclohexen-1-*yl*]-1,3-benzenediol (*O*-1602) and abnormal cannabidiol, 2 potent synthetic GPR55 agonists, have been shown to promote insulin secretion from BRIN-BD11 cells and isolated mouse islets and exert a glucose lowering effect *in vivo* (24), a response partly blunted in islets from GPR55-deficient mice (25). In rats, chronic *O*-1602 administration reduces adipocyte expression of genes encoding lipolytic enzymes, consonant with an observed increase in adiposity (26). Sustained *in vitro* incubation of adipocytes with *O*-1602 enhances intracellular lipid accumulation, suggesting that the increased adiposity triggered by *O*-1602 in rodents may be driven by direct effects of the compound on adipose tissue (26).

Although atypical cannabinoids may promote some of the above responses *via* GPR55, the primary physiologic ligand for this receptor is thought to be an endogenous bioactive lipid called lysophosphatidylinositol (LPI) (27). GPR55 is also activated by oleoylethanolamide and palmitoyl-ethanolamine, which, like anandamide, are fatty acid amides, raising the possibility that GPR55 may function as a lipid-sensing receptor. LPI and oleoylethanolamide promote insulin release from isolated mouse islets (24, 28), and in humans modulation of the LPI/GPR55 system has been positively linked with increased obesity by poorly understood mechanisms (29). The significance of GPR55 as a potential regulator of insulin action and energy homeostasis is further strengthened by the finding that GPR55^−/−^ mice exhibit insulin resistance and increased adiposity (30). Skeletal muscle, adipose tissue, and liver represent principal targets for insulin action, but it is unknown whether GPR55 influences insulin action in these tissues or whether the insulin resistance observed in GPR55^−/−^ mice are a consequence of impaired insulin action in these key metabolic tissues. The studies reported herein explore how GPR55 deficiency affects insulin action in skeletal muscle, adipose tissue, and liver and examine whether modulation of GPR55 in cultured cells that serve as empirical models of these tissues exhibit changes in insulin signaling *in vitro*.

## MATERIALS AND METHODS

Bovine insulin and LPI were purchased from MilliporeSigma (Burlington, MA, USA). *O*-1602 and *O*-1918 were from Tocris Bioscience (Abingdon, United Kingdom). Anti–insulin receptor substrate 1 (IRS-1) and phosphatase and tensin homolog (PTEN) antibodies were from Santa Cruz Biotechnology (Dallas, TX, USA). Antibodies to phospho-CREB (Ser^133^), phospho-ERK1/ERK2 (Thr^202/204^), phospho-PKB/Akt (Thr^308^), phospho-PKB/Akt (Ser^473^), phospho–glycogen synthase kinase-3 (GSK3)α/β (Ser^21/9^), phospho-FoXO1 (Thr^24^)/FoXO3a (Thr^32^), native PKB/Akt, phosphoinositide dependent kinase-1 (PDK1), FAS, perilipin, fatty acid binding protein 4 (FABP4), peroxisome proliferator-activated receptor-γ (PPARγ), Rho kinase 1 (ROCK1), myosin phosphatase targeting protein1 (MYPT1), and phospho-MYPT1 (Thr^696^) were from Cell Signaling Technology (Beverly, MA, USA). Antibodies for PGC-1α and the insulin receptor β-subunit were from Abcam (Cambridge, United Kingdom) and Merck-Millipore (Darmstadt, Germany), respectively. Anti-actin and α-tubulin antibodies were from MilliporeSigma. Horseradish peroxidase (HRP)-conjugated anti-rabbit IgG and anti-mouse IgG were from New England Biolabs (Beverley, MA, USA). The antibody targeting the catalytic subunit of protein phosphatase 2A was generated by the Division of Signal Transduction Therapy (University of Dundee, Dundee, United Kingdom). All primers were synthesized by our in-house Oligonucleotide Synthesis Service.

### Animals

Homozygous GPR55^−/−^ mice were generated by intermating heterozygous GPR55^+/−^ mice and genotyping the F1 progeny as previously described (31). Male wild-type (WT) C57BL/6J and GPR55^−/−^ mice were maintained in the University of Aberdeen Medical Research Facility at 21 ± 2°C on a 12 h light/dark cycle with *ad libitum* access to food (CRM; Special Diets Service, Essex, United Kingdom) and water. All studies were in accord with the ARRIVE guidelines (32) and performed in line with the UK Animals (Scientific Procedures) Act 1986.

### Body composition, temperature, physical activity, and metabolic parameters

Analysis of various body parameters was carried out from 10 to 22 wk of age in male WT and GPR55^−/−^ mice. Fat and lean mass were analyzed using echo resonance scanning (EchoMRI Whole Body Composition Analyzer; EchoMRI LLC, Houston, TX, USA) and expressed as percentage of body weight. For analysis of core body temperature and physical activity, transmitters were implanted intraperitoneally at 3 mo of age. Mice were allowed a 3–4 wk recovery period prior to data acquisition. Home cages were placed onto transponder energizers (ER-4000 Receiver; MiniMitter, Bend, OR, USA), allowing noninvasive monitoring of body temperature and physical activity throughout the study period. The VitalView Data Acquisition System (MiniMitter) collected data at 1-min intervals.

Energy expenditure, resting metabolic rate, and respirometry quotient were determined in 4-mo-old mice by indirect calorimetry using an Oxymax Deluxe System (Columbus Instruments, Columbus, OH, USA). Measurements were taken over 4 d, with d 1 data disregarded to allow for acclimatization. Airflow was maintained at 0.5 L/min with ambient temperature of 21 ± 1°C. Readings were taken over 10-min cycles at 2-min intervals (90 s with a 30 s purge) from each chamber plus a reference air sample per cycle.

### Blood sampling and tissue harvesting

In some experiments, blood glucose and plasma triglyceride concentrations were determined in unfed (6 h) mice using a glucometer (OneTouch Ultra Blood Glucose Monitoring System; Johnson and Johnson’s, High Wycombe, United Kingdom) or Triglyceride Quantification Kit (Abcam). Plasma insulin levels were measured using a Mouse Insulin ELISA Assay (Mercodia, Uppsala, Sweden). WT and GPR55^−/−^ mice were also studied postprandially. Animals were anesthetized with a mixture of ketamine (120 mg/kg) (Vetalar; Pfizer, Dublin, Ireland) and xylazine (16 mg/kg) (Rompun; Bayer, Dublin, Ireland) *via* intraperitoneal injection, followed by an intraperitoneal injection of insulin (2 mU/g) or vehicle (control) solution 10 min prior to collection of blood *via* cardiac puncture. Immediately upon culling, blood was sampled for glucose and tissues [skeletal muscle (gastrocnemius and soleus), white adipose tissue (epididymal, retroperitoneal, subcutaneous, omental, mesemteric), brown adipose tissue, and liver] were carefully excised, snap frozen in liquid nitrogen, and stored at −80°C. Whole blood containing heparin (20 U/ml final concentration) was centrifuged at 8000 *g* for 5 min at room temperature, after which the plasma supernatant was collected and stored at −80°C.

### Cell/tissue processing and analysis

Rat L6 myotubes, human myoblasts, murine 3T3-L1 adipocytes, rat H4IIE, and human HepG2 hepatoma cell lines were cultured and prepared for RNA extraction, RT-PCR, real-time quantitative PCR (qPCR), and immunoblot analysis as previously described (33–35). In some experiments, GPR55 was silenced in L6 muscle cells using a lentiviral short hairpin RNA (shRNA) strategy as previously described (34, 35). Forward and reverse oligonucleotides for rat GPR55 shRNA were: forward, 5′-CCGGGGAAGCATCCCCATCTACACTCTCGAGAGTGTAGATGGGGATGCTTCCTTTTTG-3′; reverse, 5′-AATTCAAAAAGGAAGCATCCCCATCTACACTCTCGAGAGTGTAGATGGGGATGCTTCC-3′ (hairpin sequences are underlined). To silence expression of GPR55 in 3T3-L1 adipocytes, cells were transfected with 50 nM of small interfering RNA (siRNA) for GPR55 (On-TargetPlus SmartPool; Dharmacon, Lafayette, CO, USA) or 50 nM of control siRNA (On-TargetPlus Nontargeting Control siRNA; Dharmacon) using JetPrime PolyPlus Transfection reagent according to manufacturer’s instructions. A list of primers used for qPCR and RT-PCR analysis is provided in **Table 1**.

**TABLE 1:** List of primers used for RT-PCR and qPCR analysis

			Primer, 5′–3′	
Gene	Species	Application	Sense	Antisense
GPR55	Mouse	RT-PCR	CTATCTACATGATCAACTTGGCTGTT	TGTGGCAGGACCATCTTGAA
GPR55	Mouse	qPCR	CCATATCCCCACCTTCCTCC	CACTCCACCAGAGTGCAGAA
GPR55	Rat	RT-PCR	TCACCATCTGCTTCATCAGC	CCTTACCAGGAACTGCAGGA
GPR55	Human	RT-PCR	TGGGCCTCTAAGAACCACAG	CCGTCCCTCCCTACATGATC
FABP4/aP2	Mouse	qPCR	GCGTGGAATTCGATGAAATCA	CCCGCCATCTAGGGTTATGA
Fasn	Mouse	qPCR	CAGCCTCCTAAGCCAGGAG	CCTCCACAGACAGGAAGATAGG
PPARγ	Mouse	qPCR	ATGCCAAAAATATCCCTGGTTTC	GGAGGCCAGCATCGTGTAGA
18S rRNA	Mouse	qPCR	CAGCCACCCGAGATTGAGCA	TAGTAGCGACGGGCGGTGTG

Animal tissues were prepared for immunoblot analysis using antibodies against proteins of interest as previously described (34, 35). Primary antibody detection was carried out using anti-rabbit IgG-HRP or anti-mouse IgG-HRP–linked antibody (New England Biolabs) as appropriate by ECL. Resulting band intensities were quantified using ImageJ software (National Institutes of Health, Bethseda, MD, USA).

### Statistical analysis

Data were analyzed using GraphPad Prism (GraphPad, La Jolla, CA, USA). Statistical analysis was performed using one- or two-way ANOVA (with Bonferroni post hoc test) or unpaired Student’s *t* test as appropriate and considered statistically significant at *P* < 0.05. ANCOVA was performed with body mass included as a covariate for analysis of energy expenditure (36).

## RESULTS

### GPR55 deficiency *in vivo* is associated with increased weight gain, adiposity, and reduced physical activity

**Figure 1:**
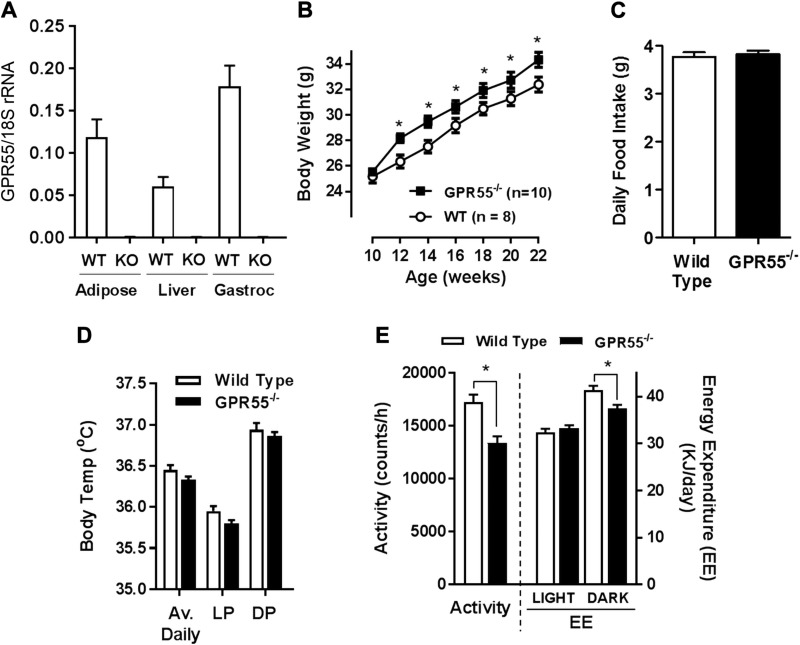
Comparison of food intake and energy expenditure in WT and GPR55-deficient (KO) mice. *A*) GPR55 mRNA expression quantified relative to that of 18S rRNA in epididymal fat tissue, liver, and gastrocnemius muscle of WT and GPR55 KO mice (*n* = 4/group). *B*–*E*) Body weight (*B*) and daily food intake (*C*) for WT and GPR55 KO mice were monitored over a 12-wk period. Body temperature (*D*), physical activity and energy expenditure (*E*) for WT and GPR55^−/−^ mice were determined along with average values in the light phase (LP) and dark phase (DP). All values presented are the means ± sem from 12 mice unless indicated otherwise. Asterisks denote statistically significant differences between WT and GPR55^−/−^ mice. *P < 0.05.

To explore the impact of GPR55 on insulin action in tissues with a key role in whole body fuel metabolism, it was important to establish that GPR55 is expressed in skeletal muscle, adipose tissue, and liver. qPCR analysis revealed that GPR55 mRNA was present in all 3 tissues from WT animals but not in those from GPR55^−/−^ (KO) mice (**Fig. 1*A***).

GPR55^−/−^ mice display no overt metabolic phenotype until 10 wk of age, after which they show a significant increase in body mass compared with WT animals (Fig. 1*B*) that cannot be attributed to increased energy (food) intake or changes in cyclical day/night body temperatures over the 10–22 wk age period (Fig. 1*C*, *D*). Analysis of physical activity revealed that GPR55^−/−^ mice exhibit a significant (22%) reduction in total daily activity relative to WT animals (Fig. 1*E*). Oxymax indirect calorimetry indicated no notable differences in resting metabolic rate or respirometry quotient between WT and GPR55^−/−^ mice (data not shown), but, in line with the reduced physical activity, energy expenditure of GPR55^−/−^ mice was significantly lower during the dark phase, a period when mice are normally most active (Fig. 1*E*).

**Figure 2:**
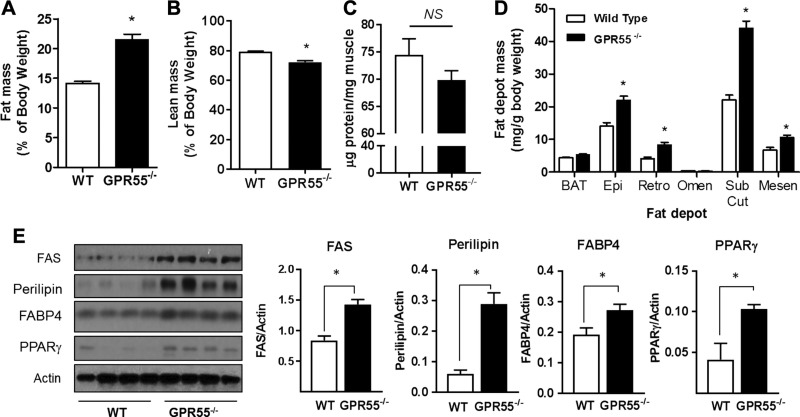
GPR55-deficient mice exhibit increased body fat mass and enhanced lipogenic drive. *A*, *B*) Mean fat (*A*) and lean masses (*B*) for WT and GPR55 KO mice at 22 wk of age were determined (*n* = 10 for WT, *n* = 8 for KO). *C*, *D*) Comparison of total protein content (per mg of muscle tissue) in gastrocnemius muscle (*n* = 6 for each group) (*C*) and mean fat depot mass (*D*) for brown adipose tissue (BAT), epididymal fat (Epi), retroperitoneal fat (Retro), mesenteric fat (Mesen), omental fat (Omen), and subcutaneous fat (Sub Cut) in WT and GPR55^−/−^ mice are presented (*n* = 12 for each group). *E*) Protein lysates prepared from epididymal fat tissue of WT and GPR55^−/−^ mice were immunoblotted using the antibodies indicated (4 individual animals/group). NS, not significant. Values presented are means ± sem. *P < 0.05.

WT and GPR55^−/−^ mice were subjected to EchoMRI analysis at 22 wk of age, and the latter exhibited a significant increase in total body fat, which was accompanied by a small but significant reduction in lean mass (**Fig. 2*A*, *B***). Analysis of total protein content expressed per milligram wet weight of gastrocnemius muscle indicated a modest (∼6%) reduction compared with WT muscle, although this failed to reach statistical significance (Fig. 2*C*). Consistent with the EchoMRI data, dissection of different fat depots confirmed significant increases in epididymal, retroperitoneal, subcutaneous, and mesenteric fat pad mass, whereas omental and brown fat depots were unaltered (Fig. 2*D*). To assess whether increased fat deposition in GPR55^−/−^ mice was underpinned by an augmented lipogenic drive, we assessed expression of key lipogenic markers. Figure 2*E* shows that expression of FAS, perilipin, FABP4, and PPARγ was significantly elevated in GPR55^−/−^ epididymal fat. We detected a significant increase in liver FAS abundance in GPR55^−/−^ mice (Supplemental Fig. 1), potentially reflecting that these animals may exhibit hepatic steatosis.

### GPR55^−/−^ mice display dysfunctional insulin signaling in adipose tissue, liver, and skeletal muscle

**Figure 3:**
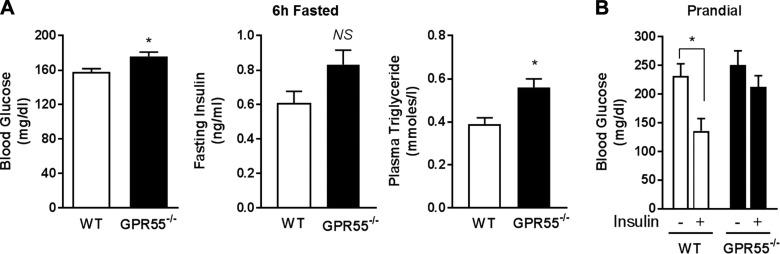
GPR55-deficient mice display elevated circulating blood glucose and plasma triglyceride levels. *A*) Blood glucose (*n* = 12 for each group), fasting plasma insulin (*n* = 5 for each group), and plasma triglyceride (*n* = 4 for each group) concentrations were determined in WT and GPR55^−/−^ mice held without food for 6 h. *B*) Prandial blood glucose levels (*n* = 3 for each group) were measured in WT and GPR55-deficient mice treated with or without insulin (2 mU/g body weight for 10 min). *P < 0.05.

Recent work has shown that GPR55-deficient mice of similar age to those used in this study are insulin resistant given that they display impaired systemic insulin sensitivity as judged on the basis of insulin tolerance analysis (30). However, this latter study did not examine the effects of GPR55 loss on insulin action in peripheral metabolic tissues. Consistent with the insulin resistance reported in these animals, GPR55^−/−^ mice held without food for 6 h exhibit modest but significant hyperglycemia and hypertriglyceridemia (**Fig. 3*A***). In addition, GPR55^−/−^ mice also display slightly increased fasting plasma insulin levels, although the difference was not significant compared with control WT animals (Fig. 3*A*). For analysis of tissue insulin action, prandial animals were acutely administered an intraperitoneal saline or insulin injection before culling, and blood and tissues were harvested 10 min postinjection. Analysis of blood glucose indicated that WT animals show a rapid reduction in blood glucose during the 10 min postinsulin treatment period (Fig. 3*B*). In contrast, although there was a tendency for blood glucose to be lowered in insulin-treated GPR55^−/−^ mice during this acute postinjection period, this reduction did not achieve statistical significance, further supporting the idea that these animals exhibit impaired insulin responsiveness.

**Figure 4:**
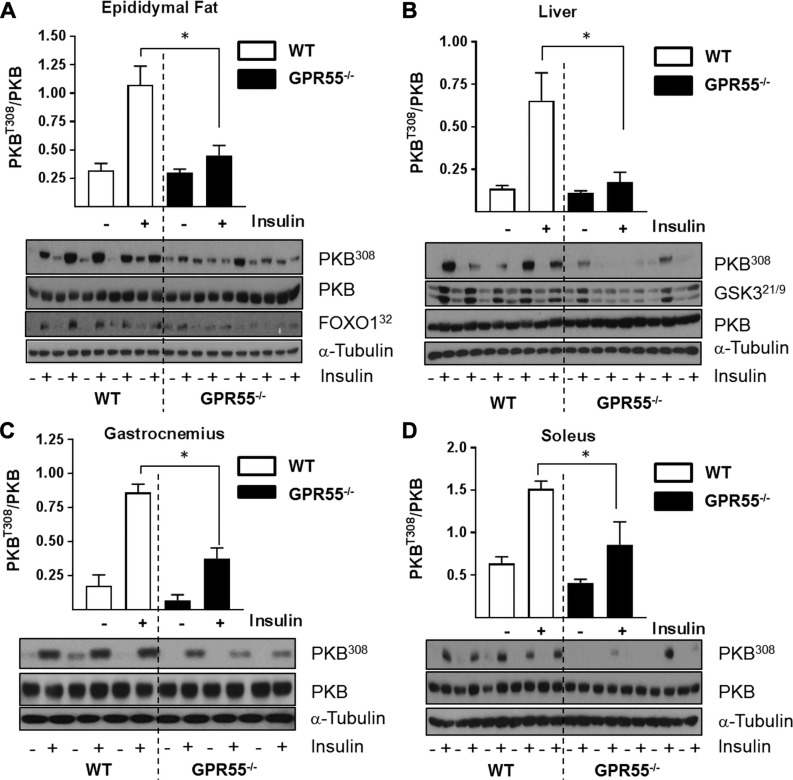
GPR55 deficiency is associated with impaired insulin signaling in peripheral tissues. Lysates prepared from epididymal fat tissue (*A*), liver (*B*), gastrocnemius muscle (*C*), and solei (*D*) of WT and GPR55^−/−^ mice stimulated with or without insulin (2 mU/g body weight for 10 min) were immunoblotted using the antibodies against phospho (Thr308) and native PKB, FOXO, GSK3, and α-tubulin as shown. Values presented are the mean ± sem from 5 individual animals. *P < 0.05.

Because adipose tissue, liver, and skeletal muscle represent major sites for insulin-mediated glucose disposal/metabolism, we assessed how insulin signaling in these tissues may be affected using PKB^Thr308^ phosphorylation as readout. Insulin induced a 3.3-, 4.8-, 4.9-, and 2.4-fold increase in PKB phosphorylation in adipose tissue, liver, gastrocnemius, and soleus muscles of WT mice, respectively (**Fig. 4**). However, the acute response to insulin in these tissues from GPR55^−/−^ mice (Fig. 4) was significantly lower by 80% (fat), 88% (liver), 55% (gastrocnemius), and 49% (soleus). PKB is also phosphorylated on Ser^473^ and, as with Thr308 phosphorylation, phosphorylation of this site was also significantly impaired in GPR55-deficient tissue (Supplemental Fig. 2). The reduced PKB phosphorylation/activation will have important consequences for regulation of its downstream targets, such as Forkhead box protein O1 (FOXO1) and GSK3, whose diminished phosphorylation (Fig. 4*A*, *B*) enhances FOXO-mediated gene transcription in adipose tissue and sustains inhibition of glycogen synthase by GSK3 in liver and skeletal muscle.

### GPR55 deficiency induces tissue specific changes in PTEN and IRS-1 expression

**Figure 5:**
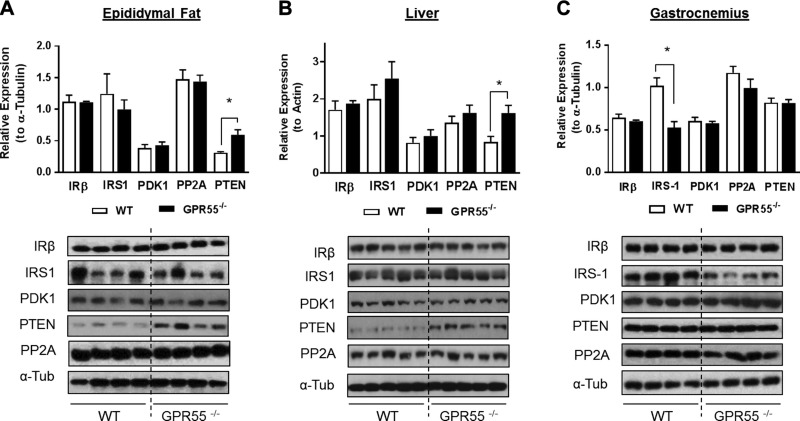
Effects of GPR55 deficiency upon key modulators of insulin signaling. Lysates prepared from epididymal fat tissue (*A*), liver (*B*), and gastrocnemius muscle (*C*) in WT and GPR55^−/−^ mice were immunoblotted using the antibodies against IRβ (insulin receptor β subunit), IRS-1, PDK1, protein phosphatase 2A (PP2A), and PTEN. Values presented are the means ± sem from 4 individual animals (*n* = 5 for liver samples). *P < 0.05.

To understand what may account for the reduced insulin signaling capacity in adipose tissue, liver, and skeletal muscle, we investigated the effects of GPR55 deficiency on the expression of molecules regulating proximal insulin signaling. There were no differences in insulin receptor (IRβ), IRS-1, PDK1, or protein phosphatase 2A (which can dephosphorylate PKB) abundance in adipose tissue or liver, but expression of PTEN (a 3′-phosphoinostide phosphatase) was increased in both tissues (**Fig. 5*A*, *B***). PTEN was unaltered in gastrocnemius muscle, which, by contrast, exhibits significant reduction in IRS-1 protein abundance (Fig. 5*C*).

### Effects of GPR55 modulation in L6 myotubes, 3T3-L1 adipocytes, and H4IIE liver cells on insulin action and lipogenic markers

**Figure 6:**
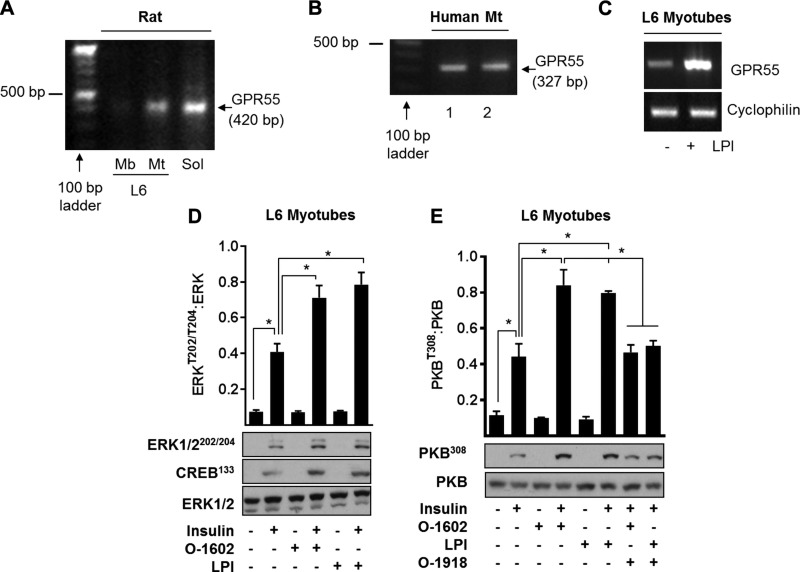
Confirmation of GPR55 expression in rat and human skeletal muscle and in cultured rat myotubes and that in myotubes modulation of GPR55 affects insulin signaling. *A*–*C*) Detection of GPR55 mRNA was performed using RT-PCR analysis in rat skeletal L6 myoblasts (Mb) (*A*) and differentiated L6 myotubes (Mt) (*A*, *C*), rat soleus (*A*), and human myotubes (*B*). The induction of GPR55 mRNA in L6 myotubes after treatment with LPI (3 µM for 48 h) was also determined by conventional RT-PCR analysis (representative of 3 independent experiments) (*C*). *D*, *E*) For insulin signaling studies, L6 myotubes were treated with 3 µM LPI, 3 µM *O*-1602, and/or 5 μM *O*-1918 for 48 h prior to stimulation with insulin (20 nM for 10 min) or vehicle control. Resulting cell lysates were immunoblotted with antibodies against phospho-ERK1/2 and phospho-CREB (*D*) or phospho-PKB^T308^ (*E*). Values presented are the mean ± sem from 3 independent experiments. *P < 0.05 between the indicated bars.

Because GPR55 deficiency is associated with impaired insulin signaling in skeletal muscle, adipose tissue, and liver, we assessed if GPR55 was expressed in clonal cell lines derived from these tissues and, if so, whether receptor activation modulates insulin-dependent signaling. GPR55 was expressed in rat soleus muscle, L6, and human myotubes (**Fig. 6*A–C***). GPR55 mRNA was barely detectable in L6 myoblasts (Fig. 6*A*), suggesting that receptor expression was likely to be differentiation linked in skeletal muscle. Pretreatment of differentiated L6 myotubes with LPI increased GPR55 mRNA, indicating that expression of GPR55 is regulated in response to its own activating ligand (Fig. 6*C*).

As expected, insulin stimulated both the MAP kinase pathway (∼4-fold) and PKB/Akt as judged by phosphorylation of ERK1/2^Thr202/Thr204^ and that of CREB^Ser133^ (which lies downstream of ERK-directed signaling) and PKB in L6 myotubes (Fig. 6*D*, *E*). Phosphorylation of these molecules by insulin was further enhanced if myotubes were pretreated with *O*-1602 and LPI (2 GPR55 agonists). Although neither agonist alone had a detectable effect on PKB phosphorylation, the ability of both ligands to enhance phosphorylation of this kinase by insulin was negated if cells were cotreated with *O*-1918, a known antagonist at GPR55 and GPR18 (Fig. 6*D*) (37).

**Figure 7:**
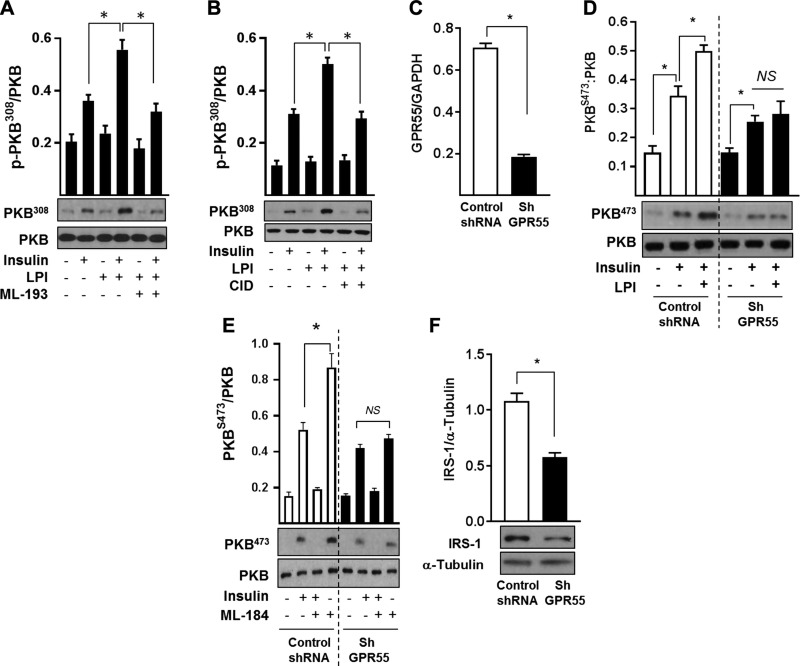
Augmentation of insulin signaling in response to GPR55 activation can be blocked by ML-193 and by shRNA-mediated receptor silencing in L6 myotubes. *A*, *B*) L6 myotubes were treated with 3 µM LPI in the presence or absence of 20 µM ML-193 (*A*) or 20 μM CID16020046 (*B*) (GPR55 antagonists) as indicated for 48 h prior to stimulation with insulin (20 nM for 10 min) or vehicle control prior to cell lysis. Resulting cell lysates were immunoblotted with antibodies against phospho-PKB^T308^ and PKB. *C*) GPR55 mRNA expression in L6 myotubes infected with lentiviral constructs containing a non-target control shRNA or GPR55 shRNA. *D*, *E*) Effects of 3 µM LPI (*D*) and 10 µM 1-(2,4-difluorophenyl)-5-[[2-[[(1,1-dimethylehyl)amino]thioxomethyl]hydrazinylidene]methyl]-1H-pyrazole-4-carboxylic acid methyl ester (ML-184) (*E*) (GPR55 agonist) on insulin signaling (phospho-PKB^S473^) in control (nontarget shRNA) infected myotubes and myotubes in which GPR55 had been silenced (GPR55 shRNA). *F*) IRS-1 protein abundance in control and GPR55-silenced L6 myotubes was determined by immunoblotting as indicated. NS, not significant. The bar values presented are the means ± sem from at least 3 independent experiments. *P < 0.05 between the indicated bars.

Given the complex pharmacology of GPR55 and the possibility that *O*-1918 may also act at GPR18, we assessed the effects of applying structurally unrelated, but selective, GPR55 antagonists *N*-[4-[[(3,4-dimethyl-5-isoxazolyl)amino]sulfonyl]phenyl]-6,8-dimethyl-2-(2-pyridinyl)-4-quinolinecarboxamide (ML-193) and 4-[4,6-dihydro-4-(3-hydroxyphenyl)-3-(4-methylphenyl)-6-oxopyrrolo[3,4-c]pyrazol-5(1H)-*yl*]benzoic acid (CID16020046) (38). As with *O*-1918, cotreatment of L6 myotubes with ML-193 or CID16020046 blocked the increase in insulin-stimulated PKB phosphorylation in response to LPI (**Fig. 7*A*, *B***). To further substantiate that these effects were indeed mediated *via* GPR55, receptor expression was stably silenced in L6 myotubes using a lentiviral-based shRNA strategy that significantly reduced GPR55 mRNA by ∼80% (Fig. 7*C*). Cells infected with a nontarget shRNA control exhibited the archetypal insulin dependent phosphorylation of PKB, which was further enhanced by pretreatment of cells with LPI. In contrast, although GPR55-depleted myotubes display a modest reduction in PKB activation/phosphorylation by insulin, they are no longer responsive to LPI (Fig. 7*D*) or to ML-184, a strong GPR55 agonist (39) that mimics the LPI response in control myotubes but not in those in which the receptor has been silenced (Fig. 7*E*). The failure of LPI or ML-184 to augment insulin-mediated PKB^S473^ phosphorylation in GPR55-depleted myotubes also excludes the possibility of GPR18 involvement. The modest decline in insulin-stimulated PKB phosphorylation that we observe in the GPR55 knockdown cells (Fig. 7*D*) may be explained, in part, by an attendant decline in IRS-1 abundance (Fig. 7*F*) and is consistent with our finding that muscle of GPR55^−/−^ mice also exhibit a reduction in IRS-1 content compared with WT muscle (Fig. 5*C*).

**Figure 8:**
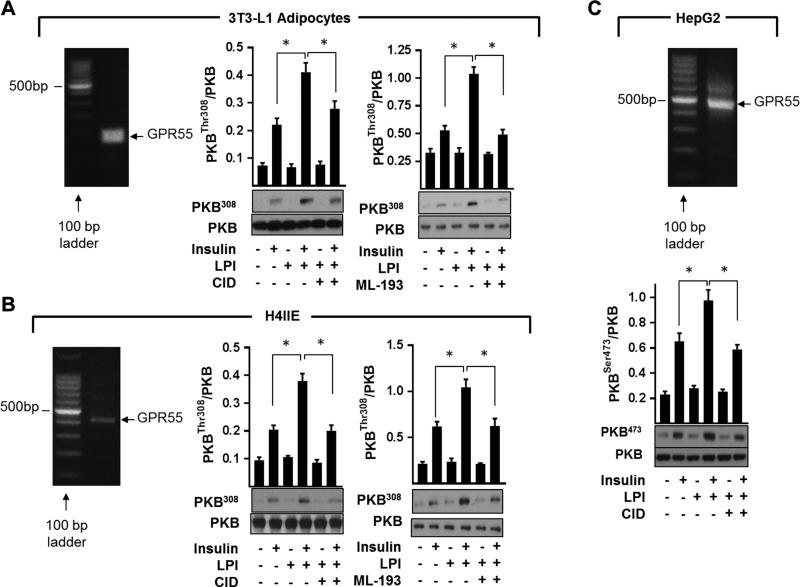
Confirmation of GPR55 expression in differentiated murine 3T3-L1 adipocytes, rat H4IIE, and human HepG2 hepatoma cells and effects of GPR55 modulation in these cells on insulin signaling. Expression of GPR55 in 3T3-L1 adipocytes (*A*), rat H4IIE liver cells (*B*), and human HepG2 hepatoma cells (*C*) was determined by conventional RT-PCR analysis (representative of 3 independent experiments). Cells were treated for 24 h with 3 μM LPI, 10 μM ML-193, and/or 10 μM CID16020046 (CID) as indicated prior to stimulation with insulin (20 nM for 10 min) or vehicle control. Resulting cell lysates were immunoblotted using the antibodies against phospho-PKB^T308^ or phospho-PKB^S473^. Values presented are means ± sem from 3 independent experiments. *P < 0.05 between the indicated bar values.

In addition to cultured myotubes, we can detect GPR55 expression in murine 3T3-L1 adipocytes, rat H4IIE liver cells, and human HepG2 liver cells (**Fig. 8**). As in myotubes, we find prior treatment of 3T3-L1 adipocytes, H4IIE, and HepG2 liver cells with LPI also enhanced insulin-dependent PKB phosphorylation; this response was effectively negated by cotreatment with the selective GPR55 antagonists CID16020046 (40) and/or ML-193 (Fig. 8).

**Figure 9:**
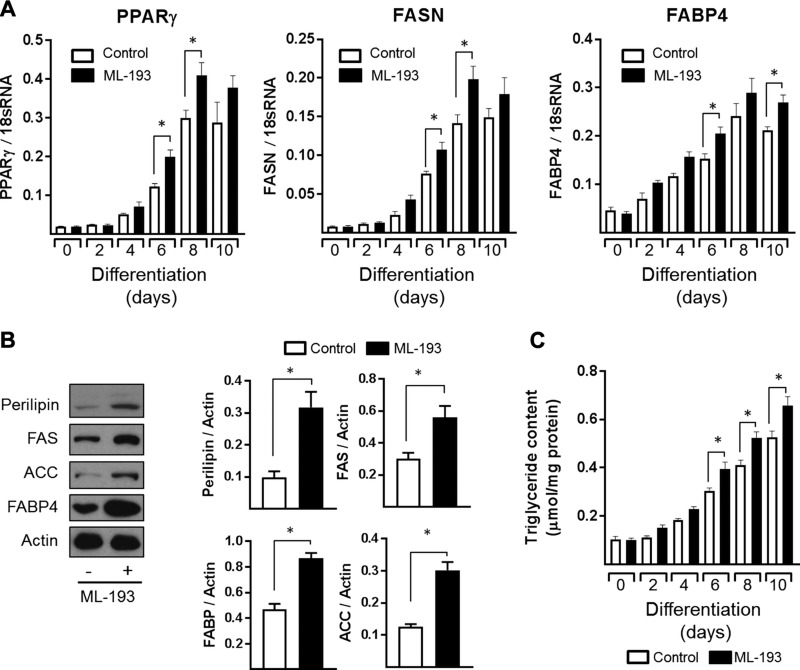
Effects of sustained GPR55 antagonism using ML-193 on adipocyte differentiation/lipogenic markers and triglyceride accumulation. Fully confluent 3T3-L1 adipocytes were allowed to differentiate for up to 10 d in the presence or absence of ML-193 (10 μM). On the indicated days, relative mRNA abundance of PPARγ, FAS, and FABP4 (*A*) as well as triglyceride (*C*) content were determined. Immunoblot analysis was performed to determine protein levels of perilipin, FAS, acetyl CoA carboxylase (ACC), FABP4, and Actin (as an internal control) in 3T3-L1 adipocytes differentiated for 6 d in the presence or absence of ML-193 (10 μM) (*B*). All quantified values presented are the means ± sem from 3 independent experiments. *P < 0.05 between the indicated bars.

**Figure 10:**
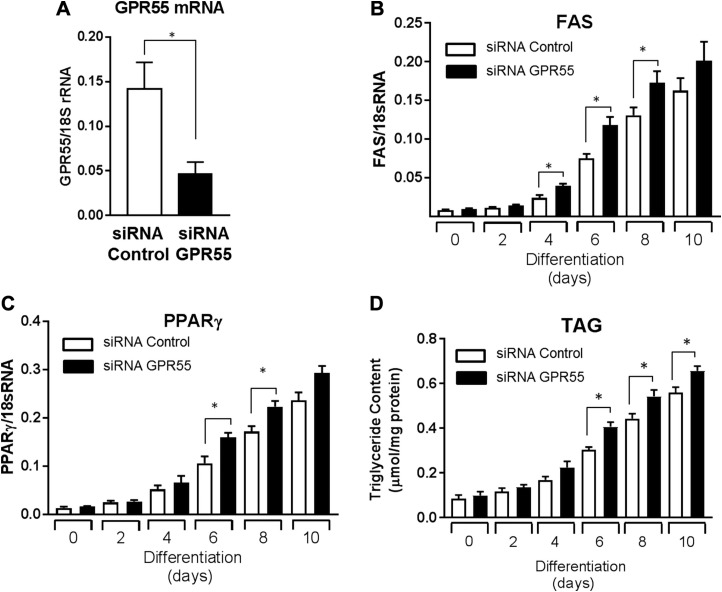
Effects of sustained GPR55 silencing on adipocyte differentiation/lipogenic markers and triglyceride accumulation. After transfection with control or GPR55-trageting siRNA, 3T3-L1 adipocytes were allowed to differentiate for up to 10 d. *A*) GPR55 mRNA abundance was determined in control and GPR55 siRNA-transfected 3T3-L1 adipocytes by qPCR analysis. *B*–*D*) On the indicated days of differentiation, relative mRNA abundance of FAS (*B*), PPARγ (*C*), and triglyceride (*D*) content were determined. All quantified values presented are mean + sem from 3 independent experiments. *P < 0.05 between the indicated bars.

We cannot exclude the possibility that the increased adiposity seen in GPR55^−/−^ mice may be a consequence of a direct loss in GPR55 action in adipose tissue rather than being linked to changes in central “brain-driven” functions. To explore this issue further, cultured 3T3-L1 adipocytes were chronically exposed to ML-193 during the course of a 10 d differentiation period to mimic a state of sustained GPR55 inactivity/deficiency. Analysis of key lipogenic markers revealed that, compared with cell cultures treated with the control vehicle solution, those chronically exposed to a selective GPR55 antagonist display elevated expression of genes encoding PPARγ, FASN, and FABP4 (**Fig. 9*A***). This ML-193–induced increase in lipogenic gene expression was associated with a significant increase in the cellular abundance of perilipin, FAS, acetyl CoA carboxylase, and FABP4 (Fig. 9*B*). The observed increase in these lipogenic/adipogenic protein markers would support greater fat accumulation, and, in line with this idea, we observed a significant increase in adipocyte triglyceride content in adipocytes treated with ML-193 (Fig. 9*C*). Consistent with these ML-193–induced responses, silencing GPR55 using siRNA (leading to ∼70% reduction in GPR55 mRNA abundance) (**Fig. 10*A***) similarly promotes significantly enhanced FAS and PPARγ (Fig. 10*B*, *C*) mRNA levels during adipocyte differentiation as well as increased adipocyte triglyceride content (Fig. 10*D*). The prolipogenic effects induced by ML-193 and GPR55 silencing in cultured adipocytes are consistent with the effects of GPR55 deficiency on adiposity *in vivo*, with further work required to establish precisely how receptor blockade mechanistically links to regulation of adipogenic genes.

## DISCUSSION

The data presented herein show for the first time that agonist-induced GPR55 activation augments insulin signaling in cultured rat myotubes, murine 3T3-L1 adipocytes, rat H4IIE, and human HepG2 liver cells and that, in rat skeletal muscle cells, GPR55 activation induces its own receptor expression. We also highlight that GPR55 deficiency in mice is associated with not only increased expression of lipogenic proteins in adipose tissue, but with significant dysfunction in insulin signaling in adipose tissue, liver, and skeletal muscle. The latter may arise, in part, through elevated expression/activity of PTEN in adipose tissue and liver and as a consequence of reduced IRS-1 expression in muscle. Precisely how PTEN abundance is up-regulated in adipose tissue and liver of GPR55^−/−^ mice is unclear, but it may involve transcripitional and/or posttranslational mechanisms (41). The PTEN gene promoter is a known target for PPARγ, which has been shown to induce PTEN expression in inflammatory and tumor-derived cells (42). Because PPARγ is up-regulated in adipose tissue of GPR55-deficient mice, this may help enhance PTEN expression. Alternatively, reduced ubiquitination/degradation of PTEN may account for its increased tissue abundance (43). Very little is known about the E3-ligases that ubiquitinate PTEN or whether they are downstream targets for GPR55 signaling. In any event, the mechanisms increasing PTEN gene expression and/or protein stability in adipose tissue and liver need not be exclusive because both would facilitate greater hydrolysis of membrane PI(3,4,5)P3 to PI(4,5)P2 and lower PKB activation at the plasma membrane (44). PTEN abundance was unaltered in skeletal muscle of GPR55^−/−^ mice, and, although the reasons for this remain unclear, it may in part be due to the much lower expression of PPARγ in skeletal muscle *vis-à-vis* adipose tissue (45). By contrast, muscle of GPR55-deficient mice has less IRS-1, which is unlikely to be accounted for by changes in gene transcription because, in separate studies, we observe no differences in IRS-1 mRNA content in WT or GPR55^−/−^ muscle (data not shown). Muscle IRS-1 abundance is known to be regulated by the ubiquitin-proteosome system (46), but whether this is influenced by GPR55-mediated signaling is not known. Nonetheless, much like the increase in adipose and liver PTEN in GPR55^−/−^ mice, loss of muscle IRS-1 reduces insulin signaling capacity (Fig. 4*C*, *D*).

The impaired insulin signaling in skeletal muscle, adipose tissue, and liver of GPR55^−/−^ mice implies that GPR55 may normally function to help sustain and/or enhance insulin action in these tissues. The finding that established cell lines that have been used extensively as *in vitro* models of these tissues express GPR55 and that *O*-1602 and LPI enhance PKB and MAP-kinase directed insulin signaling is fully congruent with this view. Moreover, the observation that the stimulatory effects of LPI on insulin signaling are lost upon silencing GPR55 expression in L6 muscle cells or upon incubation of cells with 3 structurally distinct GPR55 antagonists (*O*-1918, CID, and ML-193) provides strong evidence that GPR55 activation acts positively on insulin action in the cells we have studied. How might such regulation be exerted? GPR55 is known to couple to Gα_12/13_ and Gq proteins, which, depending on cell type and context, can potentially link to and activate multiple signaling pathways (47). Some of these pathways invoke an increase in cytosolic calcium, activation of MAP kinases, and activation of small G proteins such as RhoA, which may then act to mechanistically modulate insulin signaling components and/or key transcriptional networks, such as those regulated by CREB, NFAT, and NF-κB (47). Activation of RhoA by GPR55 would increase activity of its associated ROCK1, a Ser/Thr kinase that has been shown to support insulin signaling in skeletal muscle by promoting phosphorylation of IRS-1–Ser632/635 (48). Increased phosphorylation of these residues enhances IRS-1–PI3K association, whereas mice lacking ROCK1 not only exhibit reduced phosphorylation of these serine residues but also display impaired insulin-stimulated IRS-1–associated PI3K activity and blunted phosphorylation of PKB and that of its downstream targets, such as AS160 (48). Consistent with these findings and the observed impairment in insulin signaling in skeletal muscle of GPR55^−/−^ mice, we reveal that ROCK1 protein abundance is significantly reduced in gastrocnemius muscle of mice lacking GPR55, coinciding with reduced phosphorylation of the ROCK substrate MYPT1 at Thr696 (Supplemental Fig. 3). Whether the GPR55-RhoA-ROCK1 axis supports LPI-induced increase in insulin signaling in our *in vitro* cell models or whether loss of receptor coupling to RhoA-ROCK1 in GPR55^−/−^ mice contributes to the dysfunctional insulin signaling (alongside tissue-specific changes in PTEN and IRS-1) *in vivo* remains to be investigated.

Our observation that GPR55-deficient mice exhibit reduced physical activity and energy expenditure is consistent with the recent findings of Meadows *et al.* (30), who also reported a significant decline in spontaneous locomotor and voluntary activity in GPR55^−/−^ mice. These authors concluded that because treadmill exercise revealed no differences in exercise capacity between WT and GPR55^−/−^ mice, the decline in physical activity was more likely a consequence of reduced “brain-driven” motor functions and/or motivation rather than defects in muscle function *per se* (30). However, unlike this latter study we also detected a modest reduction in lean mass of GPR55^−/−^ mice (∼9% at 22 wk of age). The reasons for this discrepancy are unclear, but our findings imply that GPR55 signaling may normally confer a beneficial stimulus in maintaining mass of lean tissues, such as skeletal muscle and heart. It is conceivable that the reduced physical/muscular activity associated with decline in central motor function in GPR55-deficient mice serves as a pervasive, albeit indirect, atrophic stimulus (49). However, it is also plausible that, because lean tissue mass is critically dependent upon protein turnover, the impaired insulin sensitivity we observe in GPR55^−/−^ tissues such as skeletal muscle (a major component of lean body mass) may have direct effects upon protein metabolism. Insulin conveys a permissive stimulus for muscle protein synthesis and actively represses protein breakdown in this tissue (50). Consequently, reduced insulin sensitivity of GPR55-deficient muscle may contribute to a decline in its mass. The small decline in total muscle protein in the gastrocnemius is supportive of this idea. However, we have previously shown that hearts of mature GPR55^−/−^ mice exhibit a marked reduction in left ventricular wall thickness compared with age-matched WT mice, and, as such, this loss in cardiac muscle is associated with significant systolic dysfunction (51). Future studies using a targeted conditional GPR55 knockout strategy may prove instructive in helping to define the role that central and peripheral receptor expression plays in maintaining mass, integrity, and function of specific lean tissues such as skeletal muscle and heart.

Unlike the data presented herein and data reported by Meadows *et al.* (30), a more recent publication by Bjursell *et al.* (52) reported that the tendency of GPR55-deficient mice toward increased obesity was not statistically significant in their study. The reasons for this apparent incongruity remain unclear, but our analysis indicates that the increase in fat mass of GPR55^−/−^ mice cannot be attributed to greater food intake or, as others have shown (30), to changes in orexigenic and/or anorexigenic neuropeptide expression. However, a sustained reduction in physical activity and energy expenditure would be expected to shift the energy balance with time and favor fat gain with a consequential increase in body weight. The finding that increased adiposity is a feature more prominent in aged GPR55^−/−^ mice would be entirely consistent with such a proposition (52).

In addition to the reduced energy expenditure, our study reveals that increased fat mass in GPR55^−/−^ mice may also be driven by greater expression of lipogenic genes in adipose tissue potentially linking GPR55 signaling in mice as a repressor of lipogenesis/adipogenesis. This notion is further strengthened by our novel finding that chronic antagonism of GPR55 in differentiating 3T3-L1 adipocytes results in the induction of adipogenic genes and those implicated in fat synthesis/accumulation. Indeed, the demonstration that GPR55 inactivation is associated with greater triglyceride accumulation in cultured murine adipocytes is fully consistent with this observation and implies that GPR55 activity may function to help restrain increases in adiposity. However, somewhat paradoxically, GPR55 expression has been shown to positively correlate with obesity in human adipose tissue, and, although the effects of LPI on insulin signaling in human fat were not studied, LPI induced expression of lipogenic genes when incubated *ex vivo* with visceral, but not subcutaneous, fat explants (29). Moreover, in our study LPI treatment caused a slight, although not significant, reduction in lipogenic gene expression and cellular triglyceride accumulation in 3T3-L1 adipocytes during differentiation (Supplemental Fig. 4). It remains unclear whether increased expression of GPR55 in visceral fat is a cause or consequence of obesity or if the stimulatory effects of LPI on fat genes can be repressed by selective GPR55 receptor antagonists. It is difficult to reconcile why human and mouse GPR55 may impose divergent effects upon adipose tissue because no compelling evidence of differences in ligand pharmacology or receptor signaling exists among the 2 species. Indeed, this issue is further confounded by the finding that activation of human or rodent GPR55 in other cell or tissue types, such as isolated pancreatic islets or, as shown in the current study, HepG2 (a human-derived liver cell line) and H4IIE (a rat-derived cell line), can elicit very similar effects upon insulin secretion (28) and insulin signaling, respectively. Consequently, further analysis of how GPR55 agonists/antagonists specifically modify the biology and metabolism of human adipose tissue that is free of infiltrating nonfat cell types such as macrophages, which not only express GPR55 but also exhibit lipid accumulation when the receptor is activated (53), is clearly warranted for further clarification of this issue.

## CONCLUSIONS

The current study shows that GPR55 deficiency in mice is not only associated with increased adiposity, reduced physical activity, and energy expenditure but that adipose tissue, liver, and skeletal muscle exhibit a significant reduction in insulin signaling capacity, which may be a consequence of tissue-specific changes in PTEN and IRS-1 expression. The notion that GPR55 may regulate insulin action is further strengthened by our finding that receptor activation in 4 different cells lines augments insulin signaling and that this response can be negated by 3 structurally distinct GPR55 antagonists or by genetically silencing receptor expression in myotubes. Collectively, our observations indicate that therapeutic modulation of peripheral GPR55 activity may provide a means for countering obesity-linked metabolic dysfunction and insulin resistance and improving the metabolic status of such tissues.

## Supplementary Material

This article includes supplemental data. Please visit *http://www.fasebj.org* to obtain this information.

## Abbreviations

CB: cannabinoid receptor
CID16020046: 4-[4,6-dihydro-4-(3-hydroxyphenyl)-3-(4-methylphenyl)-6-oxopyrrolo[3,4-c]pyrazol-5(1H)-yl]benzoic acid
ECS: endocannabinoid system
FABP4: fatty acid binding protein 4
FAS: fatty acid synthase
FOXO1: Forkhead box protein O1
GPR55: G-protein coupled receptor 55
GSK3: glycogen synthase kinase-3
IRS-1: insulin receptor substrate 1
HRP: horseradish peroxidase
LPI: lysophosphatidylinositol
ML-193: *N*-[4-[[(3,4-dimethyl-5-isoxazolyl)amino]sulfonyl]phenyl]-6,8-dimethyl-2-(2-pyridinyl)-4-quinolinecarboxamide
MYPT1: myosin phosphatase targeting protein
*O*-1602: 5-methyl-4-[(1R,6R)-3-methyl-6-(1-cyclohexen-1-*yl*]-1,3-benzenediol
PTEN: phosphatase and tensin homolog
PPARγ: peroxisome proliferator-activated receptor-γ
qPCR: quantitative PCR
shRNA: short hairpin RNA
siRNA: small interfering RNA
WT: wild type
