# Supplementary material for: GPR55 deficiency is associated with increased adiposity and impaired insulin signaling in peripheral metabolic tissues
Source: FASEB J. 2018 Aug 27;33(1):1299–312. doi: 10.1096/fj.201800171R (PMC6355063; doi:10.1096/fj.201800171R)
Supplement: Supplementary file 1 [file fj.201800171R.sf1.pdf]

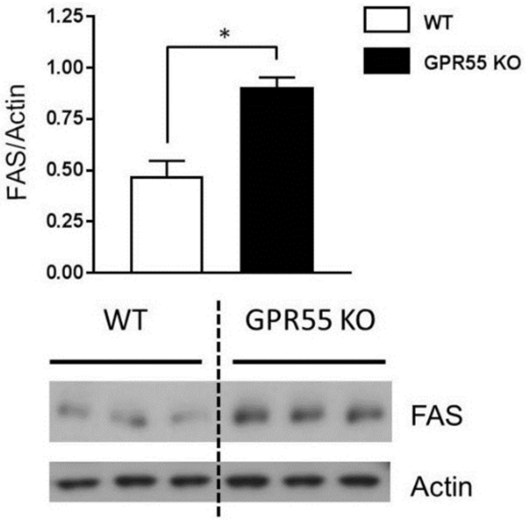

Supplementary Figure S1

Figure S1. Elevated Fatty Acid Synthase Protein Abundance in Livers of GPR55 Deficient Mice
Protein lysates prepared from epididymal fat tissue of wild type (WT) and GPR55-null (KO) mice were immunoblotted using fatty acid synthase (FAS) and actin antibodies as indicated. Values presented are the mean $\pm$ SEM from 3 individual animals. *$P < 0.05$.

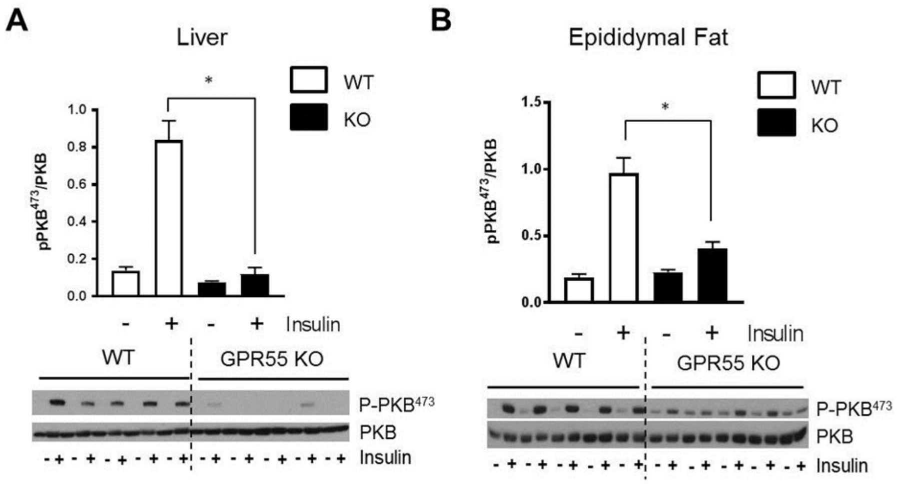

Supplementary Figure S2

Figure S2. Attenuation of Insulin Stimulated PKB/Akt Phosphorylation at Serine 473 in Liver and Epididymal Fat Tissue of GPR55 Deficient Mice

Lysates prepared from liver (A) and epididymal fat tissue (B) of wild type (WT) and GPR55-null (KO) mice stimulated with or without insulin (2 mU/g body weight for 10 min) were immunoblotted using phospho (Ser473) PKB/Akt and native PKB/Akt antibodies as indicated. Values presented are the mean $\pm$ SEM from 5 individual animals. *$P < 0.05$.

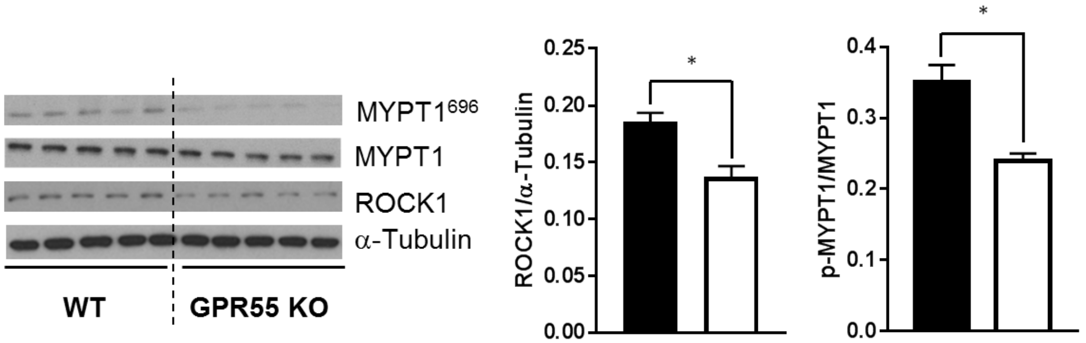

Supplementary Figure S3

Figure S3. Altered ROCK Signalling Components in Gastrocnemius Muscle of WT and GPR55 KO mice.
Protein lysates prepared from gastrocnemius muscle of WT and GPR55-deficient (KO) mice were subjected to SDS-PAGE and immunoblotting using the antibodies shown. Quantified values are the mean $\pm$ S.E.M. from 5 individual animals. * *P<0.05*.

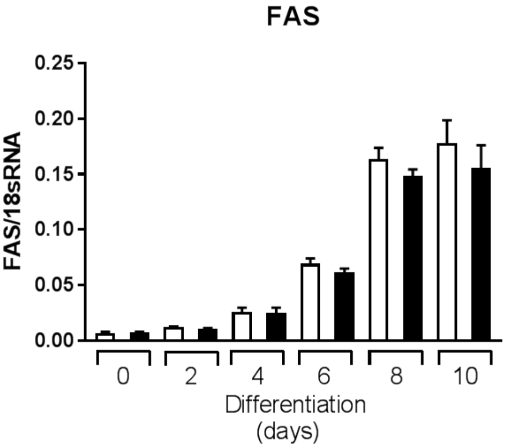

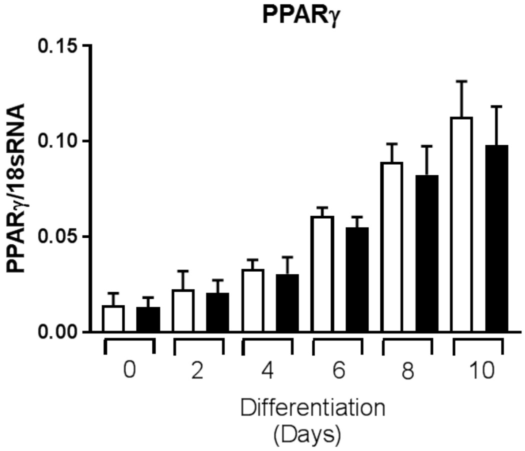

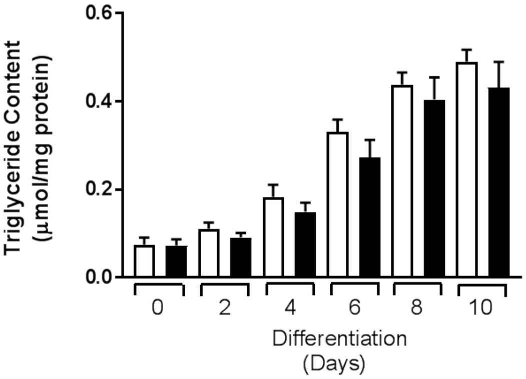

Supplementary Figure S4

Figure S4. Effects of LPI upon Adipocyte Differentiation/Lipogenic Markers and Triglyceride Accumulation

3T3-L1 adipocytes were allowed to differentiate for a period of up to 10 days whilst treated with 3 μM LPI (black bars) or vehicle control (white bars). Total RNA was extracted and used to measure relative abundance of FAS (A) and PPARγ mRNA (B) on the indicated days of differentiation by qPCR analysis. Triglyceride content was also determined on the indicated differentiation days (C). All values presented are the mean ± S.E.M. from three independent experiments. * $P < 0.05$.
